# Regulating the Acidity and Pore Structure of Hβ Zeolite with Citric Acid Concentration for Optimized Aniline Condensation Catalysis

**DOI:** 10.3390/ma19101993

**Published:** 2026-05-12

**Authors:** Lingyi Mao, Yanyao Li, Kande Liu, Naiwang Liu, Li Shi, Xuan Meng

**Affiliations:** International Joint Research Center of Green Energy Chemical Engineering, East China University of Science and Technology, Shanghai 200237, China; 13047183415@163.com (L.M.); y30220161@mail.ecust.edu.cn (Y.L.); lkdrose@163.com (K.L.); liunw@ecust.edu.cn (N.L.); yyshi@ecust.edu.cn (L.S.)

**Keywords:** aniline condensation reaction, Hβ zeolite, citric acid modification

## Abstract

**Highlights:**

- We develop a green and scalable citric acid modification method to simultaneously optimize the acidity and pore structure of Hβ zeolite;
- The study reveals the critical role of citric acid concentration in regulating aluminum species (extra-framework/framework) and acid site distribution;
- The study provides a clear structure–activity relationship for Hβ zeolite-catalyzed aniline condensation, which is valuable for industrial catalyst optimization.

**Abstract:**

Diphenylamine is an important organic chemical intermediate, and its industrial synthesis is mainly achieved through the continuous condensation of aniline. In this study, Hβ zeolite was modified with citric acid, and its catalytic performance in the aniline condensation reaction for diphenylamine synthesis was systematically investigated. The crystal structure, acidic characteristics, pore properties, and Si/Al composition of the catalysts were comprehensively characterized by means of XRD, SEM, BET, Py-IR, ICP, and ^27^Al MAS NMR. The catalytic activities of Hβ zeolites modified with different concentrations of citric acid were evaluated in a micro fixed-bed reactor, and the structure–activity relationship was systematically discussed in combination with the characterization results. The results demonstrate that the Hβ zeolite modified with 1.5 mol/L citric acid achieves precise matching with the aniline condensation reaction in terms of crystal structure integrity, pore channel parameters, acid property distribution, and Si/Al ratio regulation. Compared with the unmodified catalyst, its catalytic activity is improved by approximately 28%, with a diphenylamine selectivity of 100%. This study proposes a modification mechanism of Hβ zeolite by citric acid.

## 1. Introduction

Diphenylamine is an essential organic amine feedstock with irreplaceable value in modern industry [1], boasting extensive applications across multiple fields. Endowed with excellent antioxidant properties by its unique molecular structure, it is widely used as an antioxidant in the rubber industry and a stabilizer in lubricant and fuel additives. It also serves as a key intermediate for synthesizing high-efficiency, low-toxicity pesticides in agriculture, charge transport layer materials for organic semiconductors in electronic materials, and various pharmaceuticals and dyes. Furthermore, via nitration, halogenation, alkylation and other reactions, diphenylamine can be converted into numerous high-value-added fine chemicals, promising broad application potential in fine chemical engineering [2,3,4].

The synthesis methods of diphenylamine have undergone years of development, mainly including batch aniline condensation, continuous aniline condensation, aniline–phenol condensation, and nitrobenzene reduction. The batch aniline condensation process employs a tank reactor, where aniline undergoes condensation under acidic catalysts. Although this process is simple to operate, it suffers from low reaction efficiency, high energy consumption, and difficult catalyst recovery. While the aniline–phenol condensation method can reduce raw material costs, it tends to generate phenolic by-products during the reaction, increasing separation difficulty. The nitrobenzene reduction method is limited in industrial application due to the use of noble metal catalysts and low product selectivity [3]. In contrast, the continuous aniline condensation process utilizes a fixed-bed reactor, where intermolecular dehydration condensation of aniline (as the sole raw material) occurs over solid acid catalysts to produce diphenylamine with ammonia release. This process boasts advantages such as a simple flow, low energy consumption, easy product separation, and continuous catalyst usage, thus becoming the mainstream technology in current industrial production [5,6].

The core of the continuous aniline condensation process lies in the development of high-performance catalysts. Currently, industrial catalysts mainly include halogens and their oxides (e.g., AlCl_3_, FeCl_3_), activated clay, activated alumina, and zeolites. Despite their high activity, halogen-based catalysts cause severe equipment corrosion, difficult product separation, and significant environmental pollution. Activated clay and activated alumina catalysts have relatively large pore channels but insufficiently strong acidity. Once coking occurs, they will be deactivated with a short service life [7]. In contrast, zeolites exhibit remarkable advantages in this reaction due to their unique pore structure and tunable acidic properties. The regular pore structure of zeolites can inhibit the formation of macromolecular by-products such as polycyclic aromatic hydrocarbons through steric hindrance effects, thereby improving target product selectivity [8,9]. Their special pore system facilitates the diffusion and mass transfer of reactants and products, delaying coking formation. Meanwhile, the Brønsted acid (B acid)/Lewis acid (L acid) ratio and acid strength distribution of zeolites can be precisely regulated via modification methods such as ion exchange, dealumination, and metal loading, optimizing the catalytic reaction pathway. Among various zeolites, β zeolite has emerged as the preferred catalyst for continuous aniline condensation due to its suitable pore size (0.64 × 0.76 nm), diverse acid site types, and excellent hydrothermal stability. Hronec et al. [5]. investigated the β zeolite-catalyzed aniline condensation reaction in a closed vessel at 350 °C, and the β zeolite with a Si/Al atomic ratio of 12.5 exhibited the highest catalytic activity. Yang et al. [10]. attempted to composite β zeolite with bentonite, and the Hβ zeolite loaded with 25% bentonite achieved the optimal catalytic activity under the conditions of 320 °C, 2 MPa and a liquid hourly space velocity (LHSV) of 2 h^−1^, affording an aniline conversion of 9.9% along with excellent selectivity for diphenylamine.

The catalytic performance of β zeolite is closely related to its microstructure. Its three-dimensional 12-membered ring pore system consists of two mutually perpendicular channels: one elliptical (0.56 × 0.56 nm) and the other approximately circular (0.75 × 0.57 nm). This pore structure not only facilitates the diffusion and adsorption of aniline molecules (kinetic diameter of approximately 0.56 nm) but also provides sufficient space for the desorption of diphenylamine molecules (kinetic diameter of approximately 0.70 nm) [11]. The acidity of β zeolite mainly originates from framework aluminum atoms, featuring two types of acid sites: Brønsted acid sites (B acid) and Lewis acid sites (L acid) [12]. Studies have shown [13] that B acid sites serve as the main active sites in aniline condensation, responsible for protonating aniline molecules to form reaction intermediates; L acid sites assist in intermediate conversion through coordination, and the synergistic effect between the two significantly enhances catalytic activity. Beyond their catalytic role, the functional performance of zeolite-based materials is also strongly governed by structure–function relationships involving pore architecture, molecular adsorption, and mass transport. Recent studies on zeolite-based systems have shown that pore size, porous accessibility, and the local chemical environment can critically influence molecular recognition and transport behavior. For instance, zeolite 3A-based sensing systems have been reported to benefit from the porous structure and ion-exchange characteristics of the zeolite framework, while zeolite 5A-based platforms have demonstrated pore-size-dependent selective adsorption of target molecules [14,15]. These observations highlight that the regulation of pore environment and framework-related species is important not only for catalysis, but also for adsorption and transport processes. In this context, the modification of Hβ by citric acid in the present work may affect catalytic performance through the combined regulation of acidity, pore accessibility, and diffusion environment.

Despite these advantages, pristine β zeolite still exhibits several limitations: first, extra-framework aluminum species may exist in the pores, blocking some active sites and reducing catalyst utilization; second, the excessively high proportion of strong acid sites tends to induce deep polymerization of aniline molecules, generating coking precursors and leading to catalyst deactivation; third, the pore size distribution is not sufficiently concentrated, with some micropores being too small to hinder the diffusion of diphenylamine molecules. To address these issues, researchers have adopted various modification methods to optimize β zeolite, such as high-temperature calcination, steam treatment, acid treatment, and ion exchange [16,17]. Among these, acid treatment is widely used due to its simplicity and precise regulation [18,19,20]. As a mild organic acid, citric acid can selectively remove extra-framework aluminum and part of framework aluminum through complexation during modification without damaging the crystalline structure of the zeolite, achieving synergistic regulation of acidic properties and pore structure. Li et al. [21]. treated USY zeolite with citric acid and applied it to aniline condensation, and the citric acid-treated USY zeolite showed higher catalytic activity. FAU-type zeolites feature intrinsic supercages in their framework structure, and the large cavity space of these supercages allows for the enrichment of more aniline reactants inside. Such aniline accumulation is prone to trigger uncontrolled polyaniline condensation reactions, which further result in severe coke deposition on the zeolite surface. Compared with USY zeolite, β zeolite has no supercage structure, allowing reaction molecules to diffuse freely in multiple directions with lower mass transfer resistance. Y-type zeolites typically have a low Si/Al ratio, resulting in poor thermal stability toward the reaction; their framework is prone to collapse with notable structural damage when subjected to acid-base treatment. In contrast, β-type zeolites possess a higher Si/Al ratio, thus exhibiting a more stable framework structure after acid-base treatment. Hβ zeolite is one of the most widely used catalysts in industrial aniline condensation processes. Although acidic modification of β zeolite has been reported previously, those studies did not focus on the aniline condensation reaction addressed in the present work. In studies on catalysts for aniline condensation, acid-modified USY zeolites have also been investigated. However, due to the differences in framework structure, channel system, and aluminum distribution, the effect of citric acid treatment on Hβ cannot be directly inferred from the previously reported USY-based systems.

In this study, Hβ zeolites were modified with citric acid solutions of different concentrations. The effects of citric acid concentration on the crystalline structure, acidic properties, pore characteristics, and Si/Al composition of the zeolites were systematically investigated. The catalytic activity and stability of the modified catalysts in aniline condensation were evaluated using a fixed-bed reaction setup. Combined with multiple characterization techniques, including X-ray diffraction (XRD), pyridine adsorption infrared spectroscopy (Py-IR), Brunauer–Emmett–Teller (BET) surface area and pore structure analysis, inductively coupled plasma optical emission spectroscopy (ICP), ^27^Al magic-angle spinning nuclear magnetic resonance (^27^Al MAS NMR), and scanning electron microscopy (SEM), the structure–activity relationship between catalyst structure and performance was elucidated. The optimal modification conditions were determined, and the catalytic mechanism of Hβ zeolite in aniline condensation was reasonably inferred.

## 2. Experimental Section

### 2.1. Materials and Reagents

Hβ zeolite (Si/Al = 25) used in the experiments was purchased from Nankai University Catalyst Factory (Tianjin, China). Citric acid (analytical grade, purity ≥ 99.5%), pseudo-boehmite (industrial grade, Al_2_O_3_ content ≈ 66.7%) and nitric acid (analytical grade, concentration 65–68%) were all obtained from Sinopharm Chemical Reagent Co., Ltd. (Shanghai, China). Aniline (analytical grade, purity ≥ 99.0%) was purchased from Shanghai Lingfeng Chemical Reagent Co., Ltd. (Shanghai, China).

### 2.2. Catalyst Preparation

A certain mass of citric acid was weighed and dissolved to prepare aqueous citric acid solutions with different concentrations. The pre-calcined Hβ zeolite was mixed with the prepared citric acid solutions at a solid-to-liquid ratio of 1:10, followed by stirring in a water bath at 80 °C for 3 h. The mixture was then filtered and washed thoroughly, and dried overnight in an oven at 120 °C. The dried zeolite sample and pseudo-boehmite were weighed at a mass ratio of 4:1, homogeneously mixed, and kneaded to form a paste by dropwise addition of dilute nitric acid (5 wt%). The paste was extruded into cylindrical strips using an extruder, dried, and subsequently calcined in a muffle furnace at 550 °C for 5 h. The calcined catalyst was crushed and sieved to collect 20–40 mesh particles for further use. The Hβ zeolites modified with citric acid solutions of 0.5, 1.0, 1.5, 2.0 and 3.0 mol/L were denoted as CA-0.5, CA-1.0, CA-1.5, CA-2.0 and CA-3.0, respectively. The pristine Hβ zeolite without citric acid treatment was used as the blank control.

### 2.3. Aniline Condensation for Diphenylamine Synthesis

The catalytic performance of the prepared catalysts for diphenylamine synthesis via aniline condensation was evaluated in a fixed-bed microreactor (stainless steel, inner diameter 8 mm, length 400 mm). The reaction was conducted under the following conditions: temperature 320 °C, pressure 2 MPa, liquid hourly space velocity (LHSV) 6 h^−1^, and reaction duration approximately 8 h. The reaction products were analyzed on a Tianmei GC 7900 gas chromatograph (Shanghai Techcomp Scientific Instrument Co., Ltd., Shanghai, China) with the column temperature set at 190 °C and the detector temperature at 280 °C. Quantitative analysis of the products was carried out via the external standard method with a calibrated standard curve. Under the selected reaction conditions, diffusion limitations were carefully evaluated. A relatively high liquid hourly space velocity (LHSV) was employed to minimize external mass transfer resistance. In addition, catalyst particle size screening showed that the catalytic performance depended on particle size when relatively large particles were used, whereas the catalytic performance became nearly independent of particle size once the catalyst was reduced to a sufficiently small size range. This result indicates that internal diffusion limitations were largely eliminated under the particle size adopted in this work. The corresponding data are provided in the Supplementary Information (Figure S1).

### 2.4. Catalyst Characterization

#### 2.4.1. X-Ray Diffraction (XRD)

Crystalline structures of the as-synthesized catalysts were characterized by means of X-ray diffraction (XRD) using a D8 Advance diffractometer from Bruker AXS GmbH (Karlsruhe, Germany). Measurements were conducted under the following parameters: Cu-Kα radiation (λ = 0.15418 nm), tube voltage 30 kV, tube current 20 mA, 2θ scanning range of 5–50°, scanning rate 0.02° s^−1^ and step size 0.02°. A graphite monochromator was adopted to eliminate background interference. Before XRD testing, all samples were triturated into fine powders, loaded into glass sample holders and leveled with glass slides. The crystal structure type and crystallinity of the zeolites were ascertained from the positions and relative intensities of characteristic diffraction peaks in the acquired XRD patterns. The diffraction peaks at 2θ = 7.8°, 22.5° and 28.1° correspond to the characteristic reflections of the BEA framework structure of Hβ zeolite.

#### 2.4.2. Scanning Electron Microscopy (SEM)

Scanning electron microscopy (SEM) is founded on the interactions between high-energy incident electrons and solid substrates. When an electron beam irradiates the sample surface, the excited region generates a variety of signals, including secondary electrons, Auger electrons, characteristic and continuous X-rays, backscattered electrons, transmitted electrons, as well as electromagnetic radiation in the visible, ultraviolet and infrared regions. Concomitantly, phenomena such as electron-hole pair formation, lattice vibrations (phonons) and electron oscillations (plasmons) are induced. The physicochemical properties of the samples—including morphology, elemental composition, crystal structure, electronic structure and internal electric/magnetic fields—can be elucidated by detecting and analyzing these signals with dedicated detectors. The surface micromorphologies of the catalyst samples were characterized with a GeminiSEM 500 scanning electron microscope (Carl Zeiss, Oberkochen, Germany).

#### 2.4.3. Brunauer–Emmett–Teller (BET) Surface Area and Pore Structure Analysis

The textural properties of the samples were analyzed via the static volumetric method on a JW-BK112 adsorption analyzer (Beijing Jingwei Gaobo Science & Technology Co., Ltd., Beijing, China) at 77 K. Before each measurement, all samples were degassed in situ under high-vacuum conditions at 300 °C for 3 h to eliminate adsorbed water and surface residual impurities. The total specific surface area (SBET) was calculated by means of the Brunauer–Emmett–Teller (BET) model. The micropore surface area and micropore volume were determined through the *t*-plot method at a relative pressure of p/p0 ≈ 0.99. Nitrogen adsorption–desorption isotherms were acquired using the volumetric method, and the pore size distributions of the samples were calculated using the HK/SF method.

#### 2.4.4. Pyridine Adsorption Infrared Spectroscopy (Py-IR)

The acidic properties and the densities of Brønsted and Lewis acid sites of the catalysts were investigated by means of Fourier transform infrared spectroscopy using pyridine as a probe molecule (Py-IR) on a Nicolet iS10 spectrometer (Thermo Fisher Scientific, Waltham, MA, USA) equipped with an in situ reaction cell. The self-supported sample wafer was pretreated under vacuum at 380 °C for 2 h, followed by pyridine adsorption at 80 °C for 30 min. The sample was then desorbed under vacuum at 200 and 450 °C, and the corresponding infrared spectra were recorded. The spectra obtained after desorption at 200 and 450 °C were used to characterize the total acid sites and strong acid sites, respectively, while the weak acid sites were determined from the difference between them. The absorption bands at approximately 1540 and 1450 cm^−1^ were assigned to pyridine coordinated to Brønsted and Lewis acid sites, respectively. After baseline correction, the peak intensities (or integrated band areas) of these two bands were used for quantification. The amounts of Lewis and Brønsted acid sites were calculated according to the calibration relationships $C_{L}=373A_{L}$ and $C_{B}=990A_{B}$, respectively, where $A_{L}$ and $A_{B}$ are the absorbances of the bands at 1450 and 1540 cm^−1^. The weak acid amount was obtained from the difference between the acid amounts measured after desorption at 200 and 450 °C.

#### 2.4.5. Inductively Coupled Plasma Optical Emission Spectroscopy (ICP-OES)

The Si/Al molar ratios of the samples were quantified via inductively coupled plasma optical emission spectroscopy (ICP-OES) using an Agilent 5800 ICP-OES spectrometer (Agilent Technologies, Santa Clara, CA, USA) with a radio frequency (RF) generator operating at 40.68 MHz. The zeolite samples were completely dissolved in aqua regia, and the resulting solutions were appropriately diluted for subsequent elemental analysis.

#### 2.4.6. ^27^Al Magic-Angle Spinning Nuclear Magnetic Resonance (^27^Al MAS NMR)

^27^Al MAS NMR measurements were performed on a Bruker Avance Neo 600 spectrometer (Bruker BioSpin GmbH, Rheinstetten, Germany) at a resonance frequency of 156 MHz for ^27^Al. The corresponding experiments were carried out using a 4 mm DVT H/X double-resonance probe (Bruker BioSpin GmbH, Rheinstetten, Germany) with a sample spinning speed of 15 kHz.

## 3. Results and Discussion

### 3.1. Activity of Different Acid-Modified Catalysts

**Figure 1:**
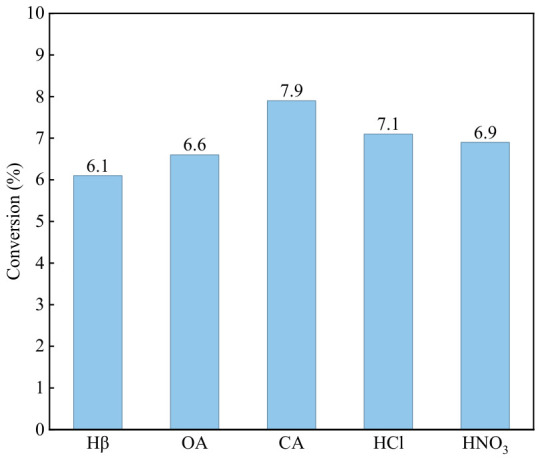
Catalytic Activity of catalysts treated with different types of acids.

Figure 1 shows the catalytic activity of Hβ zeolite treated with different kinds of acids at 2 mol/L in the catalytic condensation of aniline in a fixed-bed reactor. The conversion values in the figure were obtained after 8 h of reaction when the reaction reached a stable stage. As can be seen from the results, the four acid-modified catalysts selected in the experiment exhibited improved aniline conversion compared with the unmodified Hβ catalyst in the same aniline condensation reaction, among which citric acid afforded the best modification effect. Inorganic acids (hydrochloric acid, nitric acid) possess stronger acidity, leading to severe dealumination of Hβ zeolite during modification [22]. Although they can effectively regulate the Si/Al ratio and acidity, they tend to destroy the framework structure of the zeolite and cause excessive dealumination. In contrast, citric acid, as a mild polycarboxylic organic acid, exhibits strong chelating ability. It can preferentially remove extra-framework aluminum that blocks the pore channels, with little damage to framework aluminum [23,24]. Moreover, it can optimize the B/L acid ratio and acid distribution, thus enhancing the diffusion property and catalytic stability. Evidently, citric acid is more suitable for the acid modification of Hβ zeolite. Therefore, this study will further investigate the modification mechanism and optimal concentration of citric acid.

### 3.2. Activity of Acid-Modified Catalyst

**Figure 2:**
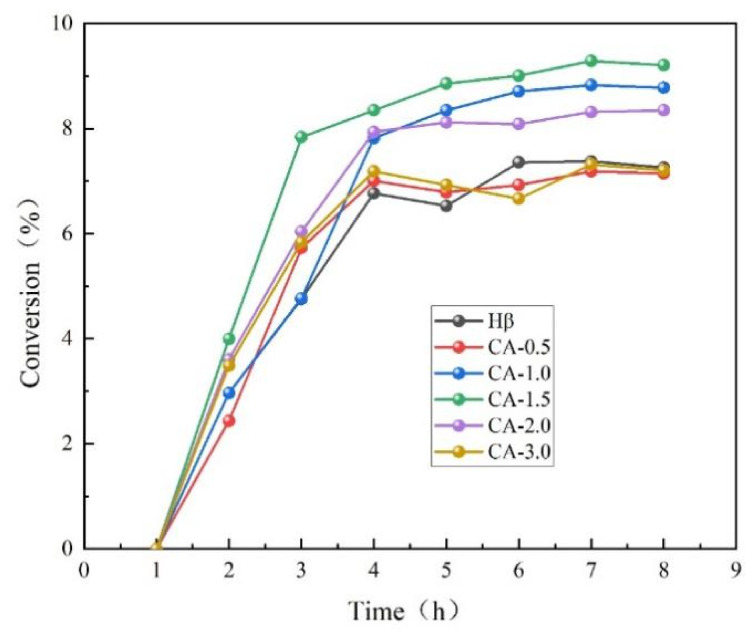
Catalytic activity of catalysts treated with different concentrations of citric acid.

A series of β zeolite catalysts modified with citric acid at different concentration gradients were prepared, and their catalytic activities were evaluated via the fixed-bed reaction of aniline condensation to diphenylamine, and the catalytic activity data are shown in Figure 2. The conversion trend after continuous reaction for 8 h under the conditions of a space velocity of 6 h^−1^, 320 °C, and 2 MPa shows that the reaction conversion rate first increases and then tends to stabilize. Based on the conversion data after stabilization in the second half of the reaction and comparison with the unmodified Hβ zeolite as a control, it can be seen that the reaction conversion rate is significantly improved after treatment with an appropriate concentration of citric acid. In the stable reaction stage, the conversion first increases and then decreases with increasing citric acid concentration, reaching a maximum at the optimal concentration of 1.5 mol/L.

### 3.3. XRD Characterization Analysis

**Figure 3:**
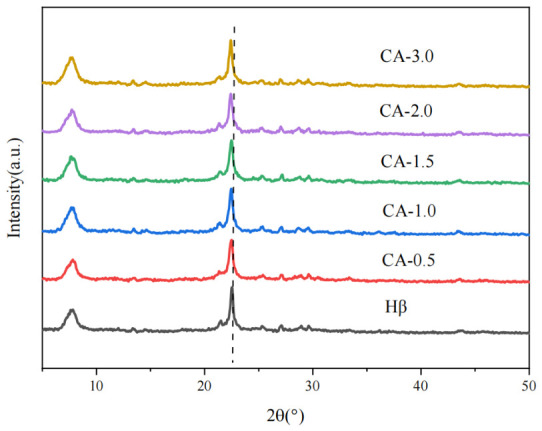
XRD patterns of Hβ zeolites modified by citric acid.

X-ray diffraction (XRD) was utilized to analyze the variations in the framework structure of the catalysts. The XRD patterns of Hβ zeolites modified with different citric acid concentrations are displayed in Figure 3. As depicted in the figure, all samples display characteristic diffraction peaks assignable to the BEA topology of Hβ zeolite at 2θ = 7.8°, 22.5°, and 28.1°, demonstrating that citric acid modification does not evidently damage the crystalline framework structure of Hβ zeolite.

Upon citric acid modification, the diffraction peak intensities of the beta zeolite exhibited a slight overall decrease, suggesting that the citric acid treatment imposes no severe destruction on the zeolite framework. However, as the citric acid concentration increased, the diffraction peaks shifted gradually toward lower 2θ angles, and the extent of this shift became more distinct at higher citric acid concentrations. This characteristic peak shift mainly reflects the removal of clogging substances in the zeolite pore structure, which relieves the lattice stress and thus induces lattice expansion [25]. As a mild organic acid, citric acid interacts with zeolite frameworks mainly via complexing and eliminating extra-framework aluminum species, without inducing significant leaching of framework aluminum or subsequent framework collapse.

### 3.4. SEM Results and Analysis

**Figure 4:**
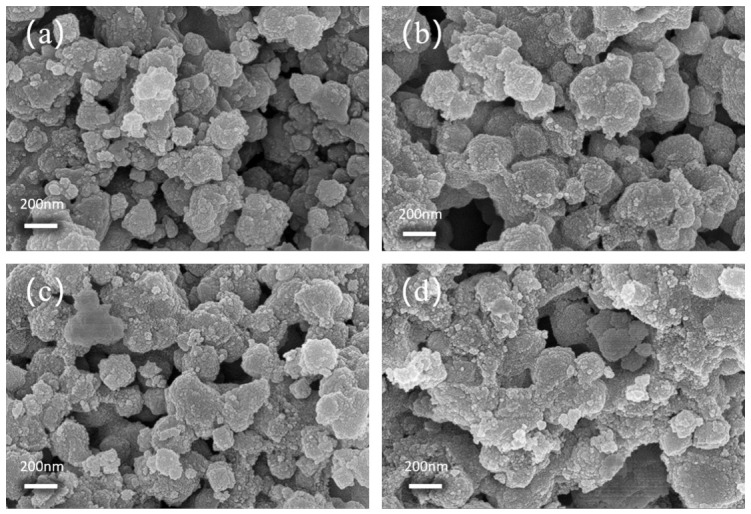
Scanning electron microscopy (SEM) images of (**a**) Hβ, (**b**) CA-0.5, (**c**) CA-1.5, and (**d**) CA-3.0 catalysts.

To investigate the micromorphology of the composite catalysts, four groups of Hβ catalyst, CA-0.5, CA-1.5, and CA-3.0, treated with different concentrations of citric acid were selected for SEM characterization. Figure 4a shows the SEM image of pristine Hβ zeolite. It can be seen from the figure that the Hβ catalyst forms aggregates composed of irregular spherical particles with a relatively uniform particle size distribution, good dispersibility, and a relatively smooth particle surface. Figure 4b–d show the SEM images of three groups of catalysts (CA-0.5, CA-1.5, and CA-3.0) treated with different concentrations of citric acid, respectively. The results indicate that the modification treatment does not significantly change the overall spherical morphology and average particle size of the particles, demonstrating that the framework structure of β zeolite is well preserved during citric acid treatment. As the citric acid concentration increased, the morphology of the zeolite catalyst gradually turned rough. After treatment with 0.5 M citric acid, the particle surface of the catalyst became smoother, accompanied by a slight increase in particle size. When the citric acid concentration was increased to 1.5 M, the particle size decreased and the smoothness of the particle surface declined. As the citric acid concentration further increased to 3 M, the catalyst particles exhibited obvious severe agglomeration, accompanied by the formation of large stacked pores. This phenomenon may be attributed to the high citric acid concentration, which causes a certain degree of framework aluminum removal and its conversion into partial extra-framework aluminum.

### 3.5. BET Characterization Analysis

**Figure 5:**
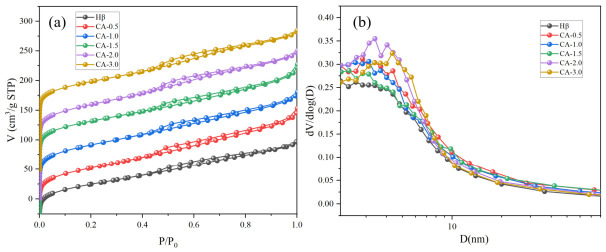
(**a**) N_2_ adsorption–desorption isotherms; (**b**) pore size distribution curves of the catalysts.

As illustrated in Figure 5a, the adsorption isotherms of the zeolite catalysts both before and after citric acid treatment belong to mixed-type isotherms, which combine the characteristics of Type I and Type IV isotherms. At low relative pressures, capillary condensation occurs in the mesopores, leading to a rapid increase in adsorption capacity. Subsequently, due to the limitation of micropore volume, the adsorption capacity gradually reaches saturation, and the isotherm tends to plateau. At high relative pressures, nitrogen molecules fill the pores through capillary condensation during adsorption; however, during desorption, the relative pressure required for the evaporation of condensed liquid in the pores is lower. Owing to the different pressure conditions between adsorption and desorption processes, the adsorption and desorption curves do not overlap, thus forming a closed loop structure. The pore size distribution profiles shown in Figure 5b suggest that all samples display highly consistent distribution tendencies, proving that citric acid modification does not change the pore types of the zeolite but only regulates the proportion of pore volume among different pore structures.

The kinetic diameter of aniline is approximately 0.56 nm, and that of diphenylamine is about 0.70 nm, enabling the product to diffuse rapidly out of the micropores [11]. As a polydentate organic ligand, citric acid forms chelates with extra-framework aluminum ions or framework aluminum in the zeolite via its carboxyl and hydroxyl groups [26,27], thereby “extracting” aluminum from the zeolite. According to the specific surface area data listed in Table 1, Hβ zeolites treated with various citric acid concentrations show noticeable differences. After treatment with 0.5 M citric acid, the acid can chelate with a portion of extra-framework or framework aluminum species in the zeolite, yet the relatively low concentration is insufficient to achieve efficient dealumination. Instead, the chelation causes partial grain agglomeration and micropore blocking, leading to a decreasing trend in micropore specific surface area. With an increase in citric acid concentration, the extra-framework aluminum clogging the pore structure is further removed, unblocking the micropore channels and thus increasing the micropore specific surface area of the zeolite. However, an inflection point occurs when the citric acid concentration exceeds 2 M, at which point the micropore specific surface area begins to decline. This may be due to the excessively high citric acid concentration, which induces slight relaxation of the framework structure and converts part of the framework aluminum into extra-framework aluminum, resulting in re-blockage of the micropore channels. The treatment of Hβ with citric acid unblocks its micropore channels, which facilitates the diffusion of reactant aniline and product diphenylamine, potentially enhancing the accessibility of active sites.

### 3.6. Pyridine Adsorption Infrared Spectroscopy Analysis

**Figure 6:**
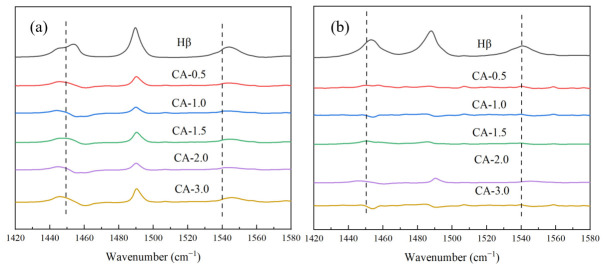
Py-IR spectra of catalysts treated with different concentrations of citric acid at (**a**) 200 °C and (**b**) 450 °C.

Pyridine adsorption infrared spectroscopy (Py-IR) was utilized to analyze the surface acidic properties of the catalyst samples. Figure 6 presents the Py-IR spectra of the catalysts treated with different concentrations of citric acid. The Py-IR spectra collected at 200 °C and 450 °C are associated with the total acid sites and strong acid sites of the catalysts, respectively. The spectral bands associated with the interactions between pyridine and Lewis (L) acid sites as well as Brønsted (B) acid sites on the sample surface are located in the wavelength range of 1400–1600 cm^−1^. Typically, the spectral bands around 1450 cm^−1^ and 1540 cm^−1^ are assigned to the stretching vibrations of pyridine adsorbed on L acid sites and B acid sites, respectively.

As listed in Table 2, in comparison with the parent Hβ zeolite catalyst, the total acid concentration of the citric acid-modified samples decreases, among which the amount of strong acid sites drops significantly. This demonstrates that citric acid can efficiently remove strong acid sites. The pristine zeolite may suffer from pore blockage (extra-framework aluminum or impurities clogging part of the micropores), leading to limited accessibility of reactants to many acid sites despite the high total acid amount. Consequently, only a small fraction of acid sites actually participate in the reaction. It should be noted that catalytic activity depends not only on the total acid amount but more critically on the accessible acid amount (effective acid amount) for reactants. Although the catalytic performance of CA-1.5 is improved, the activity cannot be simply correlated with the total acid amount measured by means of Py-IR. Citric acid treatment not only changes the number of acid sites but may also influence their spatial distribution and accessibility within the pore network. For instance, the catalyst treated with 0.5 mol/L citric acid shows a pronounced decrease in total acid amount and micropore surface area relative to the parent Hβ, while its catalytic activity does not decrease accordingly. This indicates that the catalytic behavior is not governed solely by the total density of acid sites, and that the accessibility and effectiveness of specific acid sites likely play a key role. Therefore, direct normalization of activity using the total acid amount may not accurately represent the intrinsic activity of the catalytically relevant sites in the present system. In the aniline condensation reaction, B acid sites play a dominant role. B acid protonate aniline molecules to generate highly active reaction intermediates (e.g., aniline cations) [23]. In contrast, L acid sites coordinate with the reaction intermediates to stabilize their transition states, thereby accelerating the conversion of intermediates.

Citric acid treatment can moderately remove extra-framework aluminum species, unblock micropores, expose more acid sites, generate Lewis acid sites, and preserve a considerable number of effective strong Brønsted acid sites, all of which are favorable for improving the catalytic performance of the zeolite. Nevertheless, when the citric acid concentration is excessively high (CA-2.0, CA-3.0), the amount of strong L acid sites decreases significantly, which is likely to inhibit the synergistic effect between B and L acid sites [28,29].

### 3.7. ICP Characterization Analysis

The ICP results (Table 3) indicate that citric acid treatment exerts a negligible effect on the silicon (Si) content in the zeolite, but can remove a portion of aluminum (Al). Moreover, the amount of Al removed increases with the increase in citric acid concentration, which is attributed to the chelation of citric acid with some extra-framework aluminum species, followed by their detachment from the zeolite. The maximum Al removal is achieved when the citric acid concentration is increased to 1.5 mol/L. However, an inflection point occurs when the citric acid concentration exceeds 2 mol/L, at which point the framework aluminum is partially destroyed, converted into extra-framework aluminum that aggregates and reblocks the micropore channels. The regulation of the SiO_2_/Al_2_O_3_ ratio can tune the acidity of the Hβ zeolite, and may simultaneously form an acid type distribution more favorable for the aniline condensation reaction (e.g., enhancing the synergistic effect between Brønsted acid (B-acid) and Lewis acid (L-acid) with appropriate strengths) [30].

### 3.8. ^27^Al MAS NMR

**Figure 7:**
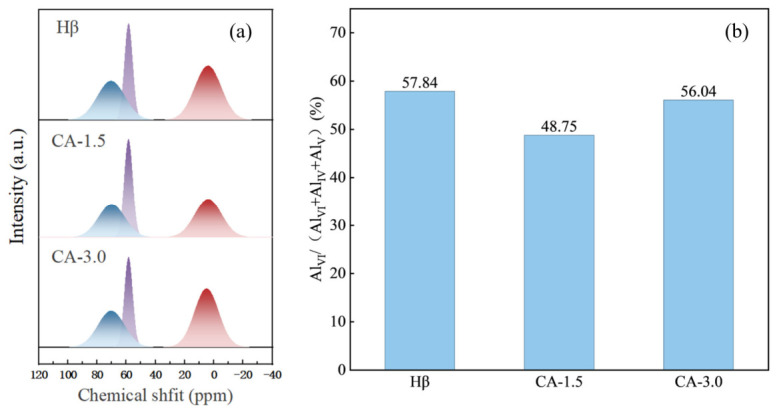
(**a**) ^27^Al MAS NMR spectra of the catalysts; (**b**) proportion of extra-framework aluminum in total aluminum.

It should be noted that the ICP-OES analysis was carried out on the shaped catalysts containing 25 wt% pseudo-boehmite binder. Therefore, the measured Al content represents the total aluminum content of the catalyst bodies rather than that of the Hβ framework alone. As a result, the Si/Al ratios derived from ICP-OES should be interpreted with caution and are used here primarily to reflect relative compositional trends among samples. To assess dealumination more reliably, the ICP results were interpreted in combination with solid-state NMR characterization. To further investigate the effect of citric acid modification on the zeolite framework, ^27^Al magic-angle spinning solid-state nuclear magnetic resonance (^27^Al MAS NMR) analysis was performed. Figure 7a presents the ^27^Al magic-angle spinning nuclear magnetic resonance (^27^Al MAS NMR) spectra of the catalyst samples. Hβ zeolite generally presents three characteristic chemical shift peaks at around 55 ppm, 35 ppm, and 0 ppm [31,32], which are assigned to framework aluminum (FAL, 35 ppm and 55 ppm) and extra-framework aluminum (EFAL, 0 ppm) [33], respectively. Hβ zeolite has a BEA topological structure with two inequivalent T-sites (Si/Al substitution sites). Differences in the Si/Al ratio, oxygen coordination environment, and framework charge distribution around Al atoms at different T-sites result in a chemical shift offset of 5–15 ppm for tetracoordinated Al [34]. The Hβ zeolite employed in this work possesses a relatively high Si/Al ratio and crystallinity, resulting in an upfield chemical shift (70 ppm) for tetracoordinated aluminum species. Consequently, the peaks corresponding to framework aluminum (FAL) appear at 55 ppm and 70 ppm.

Figure 7b presents the contents of framework aluminum and extra-framework aluminum for the blank control group, CA-1.5 group and CA-3.0 group. It should be noted that these values are semi-quantitative and are mainly used for comparative purposes. The relative proportions of tetra-, penta-, and octa-coordinated aluminum species shown in the bar chart were obtained from the peak-area contributions derived from deconvolution of the ^27^Al MAS NMR spectra. In particular, the fraction of octahedrally coordinated aluminum was calculated based on its fitted peak area relative to the total fitted aluminum signal. After citric acid treatment, the decrease in Al content in the zeolite is mainly due to the formation of water-soluble aluminum citrate chelate complexes between citrate anions and free extra-framework Al ions, which are removed by washing. However, when the citric acid concentration is excessively high, the high concentration of H^+^ dissociated from citric acid continuously attacks the Si-O-Al bridge oxygen bonds of the zeolite framework, protonating the bridge oxygen (Si-OH-Al). This causes local relaxation of the framework structure, leading to the detachment of tetracoordinated framework Al from the silica-oxygen framework and its transformation into pentacoordinated/hexacoordinated soluble Al^3+^ that enters the solution. The generated extra-framework aluminum species re-block the pore channels. According to the BET and pyridine adsorption infrared spectroscopy (Py-IR) results, samples modified with an appropriate concentration of citric acid are capable of removing the extra-framework aluminum that clogs the pore structure. This treatment not only increases the specific surface area and total pore volume of the zeolite but also modulates the acid content, thereby favoring improved contact between reactants and active sites. In contrast, when subjected to an excessively high citric acid concentration, some Si-O-Al bridge oxygen linkages are broken, which gives rise to the re-generation of extra-framework aluminum species [35]. This leads to a decline in the specific surface area and adsorption pore volume of the zeolite, and even a distinct decrease in the number of strong Lewis acid (L acid) sites. This weakens the synergistic catalytic effect between Brønsted acid and Lewis acid sites, thus degrading the catalytic activity of the zeolite.

Combining the characterization results of catalyst morphology, specific surface area and acidity, it was found that at a citric acid treatment concentration of 0.5 M, the acid only chelated with a portion of aluminum species. However, the low concentration failed to effectively remove the extra-framework aluminum, which instead aggregated in the micropore channels and caused channel blockage, thereby impairing the accessibility of acidic sites. Consequently, the catalytic activity of the CA-0.5 catalyst showed no significant improvement compared with the blank control group. With the increase in citric acid treatment concentration, the chelates formed by citric acid and part of the extra-framework aluminum clogging the micropore channels could be effectively removed, which significantly increased the micropore specific surface area of the catalyst and thus greatly enhanced its catalytic activity. The aniline conversion rate was increased by approximately 30% relative to the acid-untreated group. Nevertheless, when the acid concentration was excessively high (exceeding 2 M), the stable tetracoordinated framework aluminum in the zeolite framework structure began to be partially converted into soluble aluminum ions, which re-blocked the micropore channels. Notably, from the results of catalyst acid amount characterization, the amount of strong Lewis acid (L-acid) dropped sharply when the citric acid treatment concentration exceeded 2 M. Given that strong L-acid plays an important auxiliary role in the aniline condensation reaction, this may be another reason for the subsequent decline in catalyst activity after high-concentration citric acid treatment.

### 3.9. Reaction Mechanism

**Figure 8:**
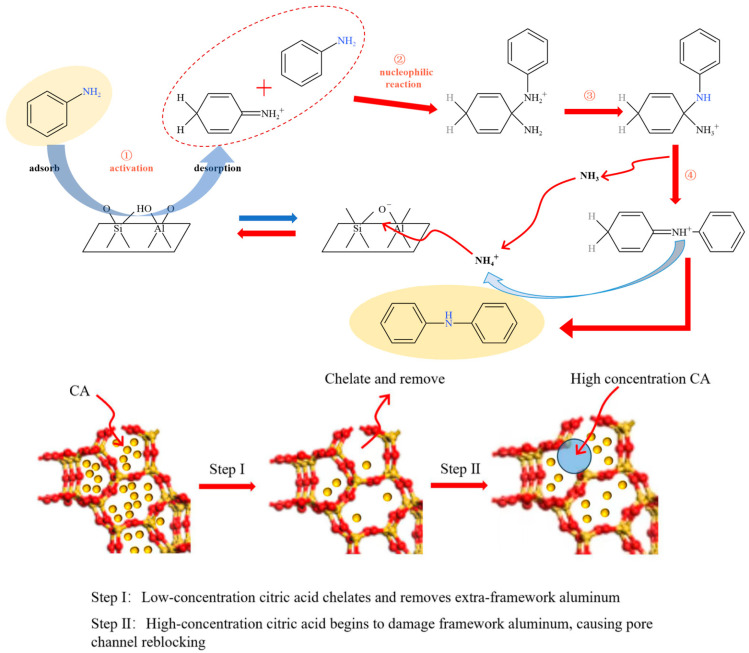
Catalytic mechanism.

The mechanism of aniline condensation and the modification mechanism of citric acid are shown in Figure 8. On the Hβ zeolite catalyst, Brønsted acid and Lewis acid sites likely play complementary roles in the condensation of aniline, with Brønsted acid sites serving as the primary active centers. The reaction is proposed to begin with protonation and activation of aniline on Brønsted acid sites, generating a more reactive aniline-derived intermediate. Subsequently, a second aniline molecule attacks the activated intermediate, leading to C–N bond formation. This is followed by proton-transfer and ammonia-elimination steps to produce diphenylamine and regenerate the acid site [6,21]. Lewis acid sites may assist the reaction by stabilizing adsorbed intermediates and facilitating the elementary transformation steps, rather than acting as the dominant active sites.

Citric acid treatment affects the catalytic performance mainly through regulation of the zeolite microenvironment. At an appropriate treatment concentration, citric acid can selectively remove extra-framework aluminum species located in the channels, thereby improving pore accessibility without significantly damaging the zeolite framework. Since acid sites are distributed both on the external surface and along the inner channel walls of the zeolite, the removal of channel-blocking species enables aniline molecules to more effectively access catalytically relevant acid sites within the micropores. Considering the favorable match between the pore size of Hβ and the molecular dimensions of aniline and diphenylamine, the improved accessibility of intracrystalline acid sites is considered an important reason for the enhanced catalytic activity after suitable citric acid modification. In contrast, excessive acid treatment may remove too many strong acid sites and deteriorate the local pore environment, leading to a decline in catalytic performance.

## 4. Conclusions

In the present work, Hβ zeolite was adopted as the catalyst and subjected to modification with citric acid of varying concentrations. By means of a series of characterization tests and comprehensive systematic analyses, the impacts of citric acid concentration on the physicochemical characteristics of Hβ zeolite and its catalytic activity in the diphenylamine synthesis reaction through aniline condensation were thoroughly explored. It was verified that citric acid modification serves as an effective optimization strategy for tailoring Hβ zeolite to the aniline condensation reaction. Essentially, citric acid modification of Hβ zeolite realizes the regulation of the catalyst’s acidity and pore channel structure. As a weak organic acid, citric acid does not damage the integrity of the crystalline structure of Hβ zeolite at the experimental concentrations; instead, its carboxyl and hydroxyl groups form chelates with extra-framework aluminum ions or framework aluminum species in the zeolite, which enables the removal of aluminum from the zeolite. This further unblocks the pore channels and increases the accessibility of acidic sites. The results show that the catalyst modified with 1.5 M citric acid exhibits the optimal catalytic activity, with the aniline conversion rate increased from 7.38% to 9.29%.

## Figures and Tables

**Table 1:** Pore structure parameters of Hβ zeolites treated with different concentrations of citric acid.

Catalyst	S_Bet_ (m^2^/g)	S_Micro_ (m^2^/g)	S_Meso_ (m^2^/g)	V_Bet_ (cm^3^/g)	PD (nm)
Hβ	606	434	172	0.359	2.37
CA-0.5	594	390	204	0.384	2.58
CA-1.0	637	436	201	0.388	2.44
CA-1.5	641	454	187	0.394	2.46
CA-2.0	613	390	223	0.386	2.52
CA-3.0	571	370	201	0.365	2.56

**Table 2:** Acid property data of Hβ zeolites treated with different concentrations of citric acid (10^−4^ mol/g).

Catalyst	Bronsted Acid Amount			Lewis Acid Amount			Total B/L
	Total	Strong	Weak	Total	Strong	Weak	
Hβ	36.71	22.55	14.16	16.02	10.53	5.49	2.29
CA-0.5	9.61	1.91	7.70	4.93	1.71	3.22	1.95
CA-1.0	8.26	1.16	7.10	2.40	0.40	1.99	3.44
CA-1.5	11.32	1.26	10.06	4.49	2.09	2.40	2.52
CA-2.0	7.64	1.91	5.73	3.95	0.11	3.84	1.93
CA-3.0	13.91	1.31	12.59	5.69	0.22	5.47	2.45

**Table 3:** Si, Al contents and Si/Al ratios of Hβ zeolites treated with different concentrations of citric acid.

Catalyst	Si (mg/L)	Al (mg/L)	SiO_2_/Al_2_O_3_
Hβ	32	10.2	6.05
CA-0.5	31	9.8	6.10
CA-1.0	33	8.4	7.58
CA-1.5	33	8.0	7.96
CA-2.0	31	9.0	6.64
CA-3.0	31	9.1	6.57

## Data Availability

The original contributions presented in this study are included in the article/Supplementary Material. Further inquiries can be directed to the corresponding author.

## Supplementary Materials

The following supporting information can be downloaded at: https://www.mdpi.com/article/10.3390/ma19101993/s1, Figure S1: Effect of catalyst particle size on aniline conversion as a function of time on stream.
