# Supplementary material for: Regulating the Acidity and Pore Structure of Hβ Zeolite with Citric Acid Concentration for Optimized Aniline Condensation Catalysis
Source: Materials (Basel). 2026 May 12;19(10):1993. doi: 10.3390/ma19101993 (PMC13208990; doi:10.3390/ma19101993)
Supplement: Supplementary file 1 [file materials-19-01993-s001.zip › materials-4242267-supplementary.pdf]

S1. Evaluation of catalyst particle size effect and diffusion limitation

To evaluate the possible influence of intraparticle diffusion, catalytic tests were carried out using Hβ catalysts with different particle-size ranges in a fixed-bed reactor at 320 °C, 2 MPa, and a liquid hourly space velocity (LHSV) of 6 h^{-1}. As shown in Figure S1, the aniline conversion increased markedly with decreasing catalyst particle size when relatively large particles were used (0–5, 5–10, and 10–20 mesh), indicating that internal diffusion limitation was significant in these cases. However, when the particle size was further reduced to 20–40 mesh, the conversion approached a plateau, and only negligible changes were observed upon further decreasing the particle size to 40–60 mesh and 60–80 mesh. This result suggests that the intraparticle diffusion resistance had been largely minimized in the 20–40 mesh range. Therefore, the 20–40 mesh fraction was selected for the catalytic evaluation in this work.

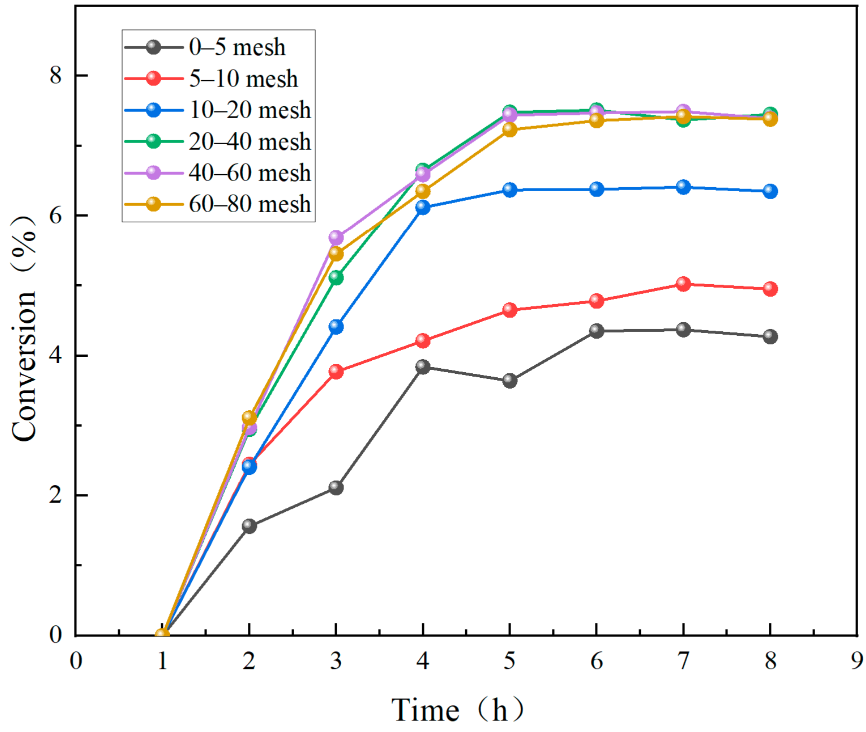

Figure S1. Effect of catalyst particle size on aniline conversion as a function of time on stream.
